# The process of building the priority of neglected tropical diseases: A global policy analysis

**DOI:** 10.1371/journal.pntd.0008498

**Published:** 2020-08-12

**Authors:** Nathaly Aya Pastrana, David Beran, Claire Somerville, Olivia Heller, Jorge C. Correia, L. Suzanne Suggs

**Affiliations:** 1 BeCHANGE Research Group, Institute for Public Communication, Università della Svizzera italiana, Lugano, Switzerland; 2 Division of Tropical and Humanitarian Medicine, University of Geneva and Geneva University Hospitals, Geneva, Switzerland; 3 Swiss School of Public Health+, Zurich, Switzerland; 4 Gender Centre, Graduate Institute of International and Development Studies, Geneva, Switzerland; Rollins School of Public Health, UNITED STATES

## Abstract

The global burden attributed to Neglected Tropical Diseases (NTDs) is 47.9 million Disability-Adjusted Life Years (DALYs). These diseases predominantly affect disadvantaged populations. Priority for NTDs has grown in recent years, which is observed by their inclusion in the sustainable development goals (SDGs). This study analyzed the process that allowed these diseases to be included on the global health policy agenda. This global policy analysis used the Shiffman and Smith framework to understand the determinants of global health political priority for NTDs. The framework comprises four categories: actor power, ideas, political contexts, and issue characteristics. Global documents and World Health Assembly (WHA) resolutions were examined, key-informant interviews were conducted, and academic publications were reviewed to understand the four categories that comprise the framework. A total of 37 global policy documents, 15 WHA resolutions, and 38 academic publications were examined. Twelve semi-structured interviews were conducted with individuals representing different sectors within the NTD community who have been involved in raising the priority of these diseases. This study found that several factors helped better position NTDs in the global health agenda. These include the leadership of actors that mobilized the global health community, the creation of a label combining these diseases as a group to represent a larger disease burden, the presence of mechanisms aligning the NTD community, and the agreement on ways to present the NTD burden and potential solutions. The process of building the priority of NTDs in the global health agenda shows that several determinants led to positive outcomes, but these diseases continue to have low priority at the global level which requires the implementation of actions to increase their global priority. These include sustaining the commitment of current actors and engaging new ones; increasing the attention given to diseases formerly categorized as “tool-deficient”, including zoonotic NTDs; continue leveraging on policy windows and creating favorable policy moments to sustain commitment, as well as setting realistic targets. Findings from this study can help develop strategies to build the momentum and drive actions to implement the goals of the new Roadmap for NTDs in the pathway to universal health coverage (UHC) and sustainable development.

## Author summary

Neglected tropical diseases (NTDs) affect vulnerable populations living mostly in tropical and subtropical settings, and disproportionately affect women and children. Historically, the priority given to NTDs in global health policies has been lower than that of HIV, malaria and TB, but in recent years it has increased. To understand the processes that helped raise the positioning of NTDs in global policies, this study used a framework by Shiffman and Smith that assembles determinants of political priority under four categories: actor power, ideas, political contexts, and issue characteristics. A total of 37 global policy documents, 15 WHA resolutions, 38 academic publications, and findings from 12 semi-structured interviews with individuals representing different sectors within the NTD community, were analyzed using a policy framework proposed by Shiffman and Smith. This study found that elements that helped increase the priority of NTDs in global policies included the presence of leaders, institutions and guiding documents to mobilize the community, the creation of the NTD label, and the way the burden and solutions were presented. To continue raising the profile of NTDs at the global level, the study presents suggestions that are in line with UHC and SDG targets.

## Introduction

Neglected tropical diseases (NTDs) are diseases of poverty that affect disadvantaged populations living predominantly but not exclusively, in tropical and subtropical environments [1]. The estimated burden of NTDs is about 47.9 million Disability-Adjusted Life Years (DALYs) [2], similar to that of tuberculosis (TB) [2,3]. Women and children are disproportionately affected by many of these conditions [4]. Consequences of these diseases include stigma, social exclusion, disability, and other chronic conditions [2,5]. Their effects inhibit the social and economic development of affected populations and nations [6,7]. Despite their high burden and long-term effects [2,5], this category of diseases has been neglected and absent from the global health agenda when compared to other infectious and tropical diseases such as HIV, malaria, and TB [1,8].

However, the global political priority of NTDs has grown in recent years. Overlooked in the Millennium Development Goals (MDGs) where they were categorized as “other diseases” [8,9], NTDs have now been acknowledged as a barrier to development [10–13] and listed in the United Nations Agenda for Sustainable Development, in goal 3.3 that states: “By 2030, end the epidemics of AIDS, tuberculosis, malaria and neglected tropical diseases and combat hepatitis, water-borne diseases and other communicable diseases” [14].

Prioritization of issues on the global health agenda is often political [15,16]. According to the Shiffman and Smith framework [15], “global political priority is the degree to which international and national political leaders actively give attention to an issue, and back up that attention with the provision of financial, technical, and human resources that are commensurate with the severity of the issue” [15]. The framework comprises four categories: actor power, ideas, political contexts, and issue characteristics; grouping 11 factors or determinants of political priority. Actor power can be understood as the strength of those with interest in the issue and their capacity to drive collective action. Ideas relate to how the issue is understood within the community and how it is portrayed among external audiences to gain attention. The political context refers to the environments and conditions within which relevant actors operate. Lastly, issue characteristics consist of how the severity of the issue and possible solutions are presented [15].

The Shiffman and Smith framework [15] has been used in studies that have investigated how global health initiatives such as maternal mortality [15], early childhood development [17], global surgery [18], and non-communicable diseases [19] have gained traction on the global agenda. The processes by which NTDs have gained attention at the policy level is less well understood, although clear milestones in the history of NTDs are evident [11,20–23]. To understand the processes that led to the increased attention of NTDs at the global level, this study examines the factors that have played a role on building the priority of NTDs, using the Shiffman and Smith [15] framework of determinants of global health political priority.

## Methods

### Data collection

**Fig 1:**
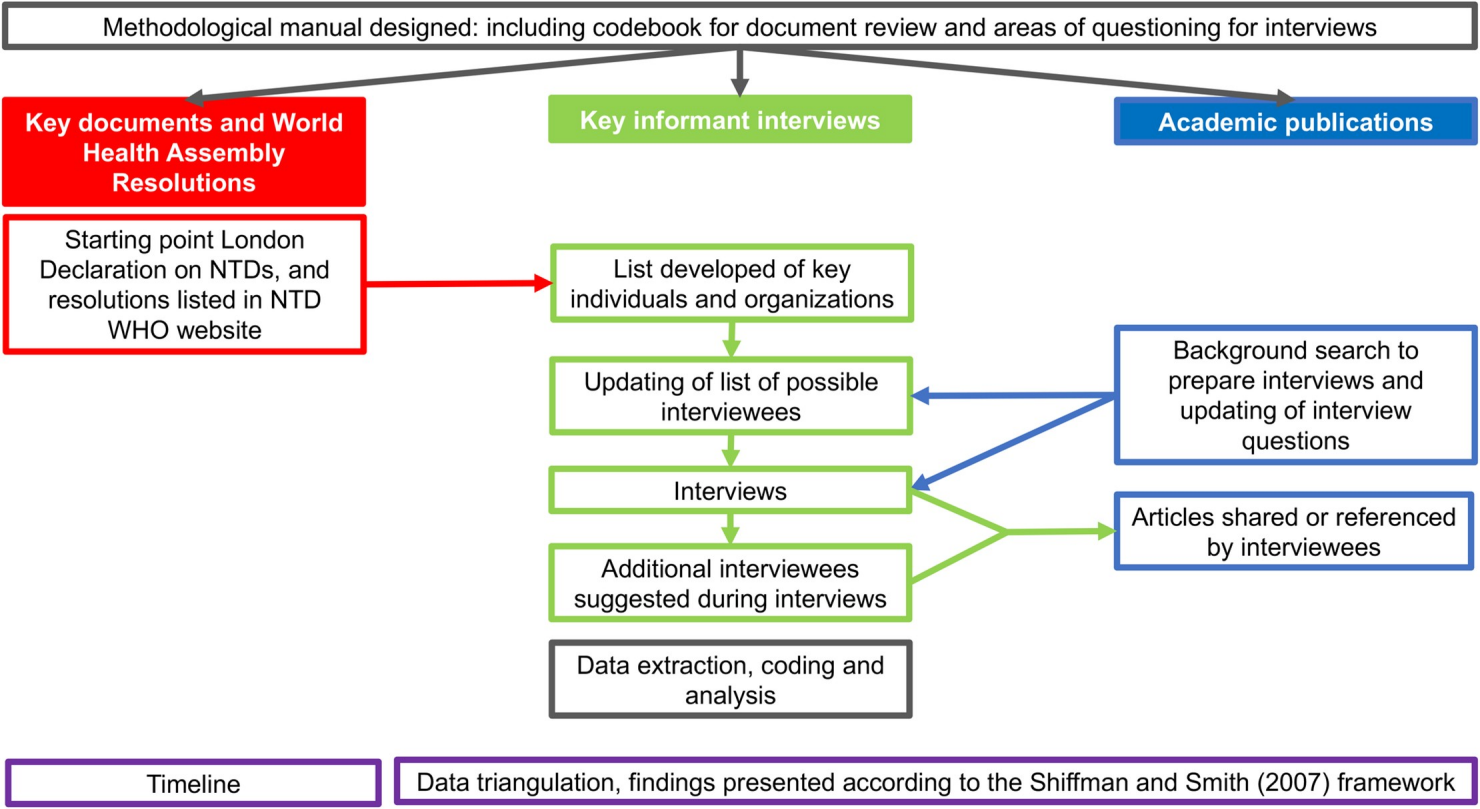
Methodological process.

To understand the four categories of determinants of global health political priority, we examined key global documents and World Health Assembly (WHA) resolutions, undertook key-informant interviews, and reviewed academic publications (see Fig 1). A similar approach was adopted for the analysis of the non-communicable disease (NCD) policy context [19].

### Analysis of key global documents and WHAs

A qualitative analysis of key global policy documents and WHA resolutions was conducted from July to December 2016. The London Declaration on NTDs [24] was selected as the entry point for the search because it was an important milestone in the process of raising the priority of NTDs globally. In conjunction, other policy documents and reports available on the websites of Uniting to Combat NTDs and the WHO were analyzed. The website of the Global Network on NTDs, a platform that advocated for NTDs, was also reviewed to identify other global policy documents used to raise the priority of these diseases at the global level [25]. This website listed G7/G8 commitments made at specific meetings which included the names of the documents where these diseases had been featured. Those documents were later retrieved for examination. Additionally, the WHA resolutions were retrieved from the WHO NTD website [26].

Two reviewers analyzed the documents (JC, NAP). One reviewer extracted data for a document, and the other reviewer cross-checked for accuracy and to ensure reliability. A codebook guided the data extraction process. Reports were produced on an ongoing basis, and preliminary findings as well as data extraction process were discussed in meetings with the co-authors of this paper. As a result of this analysis, a list of stakeholders and a timeline with key milestones in the history of NTD prioritization were developed. Both the timeline and the list of stakeholders informed the key informant interviews.

### Key informant interviews

The second component of this study took place between June 2017 and February 2018 and consisted of interviewing individuals from organizations identified during the analysis of documents. This process was carried out by one of the researchers who participated in the document analysis (NAP). Confidential semi-structured interviews with individuals representing the diversity of actors of the NTD community were conducted. The interview was organized along five themes: 1) an introduction to build rapport and contextualize the interview which included identifying the role of the interviewee in influencing health policies for NTDs, 2) questions to understand the process of policy development, 3) questions to identify key actors and their roles, 4) questions inquiring about expectations on the future of the NTD policy agenda, and 5) suggestions of other individuals to contact which facilitated snowball sampling. Examples of questions used during the interviews are available in S1 Text.

Interviews were conducted in person, by phone, or by Skype, were audio-recorded and transcribed verbatim. To ensure the anonymity of participants, a code was used to name the interview transcriptions. Analysis of the data was done deductively using the Shiffman and Smith framework of factors determining political priority which comprises four categories: actor power, ideas, political contexts, and issue characteristics [15]. Inductive analysis was also done by coding for new themes that emerged from the data. As the interviews progressed, the timeline was refined.

### Academic publications

While a literature review informed this study design and conduct, the study included the identification of academic publications through search engines to prepare for the interviews, with a focus on publications by the interviewees and the organizations they represented. In addition, articles shared by some of the interviewees before or after their interviews were included. All publications were screened to identify if they provided additional information that could help in understanding the NTD prioritization processes, more specific information related to any of the following were searched for: mentions of key documents, actors or moments in raising the priority of NTDs at the policy level. These publications helped fill in gaps from the document review and interviews, and to complement the timeline.

### Ethical approval

This study was carried out as part of a research for development project, COHESION Project [27], addressing the double burden of non-communicable and neglected tropical diseases in low- and middle-income (LMIC) countries. The study was approved by the Commission Cantonale d’Éthique de la Recherché Genève (CCER) in August 2016 (project #2016–01242).

## Results

A total of 37 global policy documents and 15 WHA resolutions were analyzed. Thirty-eight academic publications were examined. The list of documents and academic publications analyzed is provided in S2 Text.

Twelve semi-structured interviews were conducted, 11 by the lead author of this paper (NAP) and one by another researcher mentioned in the acknowledgments, ten were carried out in English and two in Spanish. Of the 12 interviewees, four were women and eight were men. Interviewees were employed at academic institutions (n = 5), non-governmental organizations (NGOs) (n = 2), non-profit organizations (NPOs) (n = 1), philanthropic foundations (n = 1), multilateral organizations (n = 1), and pharmaceutical companies (n = 2). Some of these interviewees were based in endemic countries (n = 2), others had travelled extensively or lived in those settings (n = 5), and most had previous or ongoing NTD projects with stakeholders based in LMICs (n = 10), which allowed them to bring the perspectives from the Global South. All interviewees had participated in multi-partner NTD initiatives at a global level, and some had collaborated with the WHO NTD Department (n = 6). On average, the interviews lasted one hour.

### Actor Power

The first category of the Shiffman and Smith framework [15] is actor power and refers to the strength that individuals and organizations interested in the issue have. This is portrayed by four factors: the cohesion of the policy community, the presence of leaders uniting the community, the existence of guiding institutions, and the mobilization by civil society. NTD control initiatives have been present since the 1970s and foster exchange among actors from different sectors [28]. Interviewees mentioned that at the global policy level, the number of actors has increased and the sectors they represent has varied over the years, particularly for diseases controlled with preventive chemotherapy. These include actors from endemic countries, for example, private sector companies (e.g. EMS from Brazil) or Foundations (e.g. Mundo Sano Foundation from Argentina) donating medicines [29]. The variety of actors who have joined efforts to combat these diseases [20,30], and the progress made to date to alleviate their burden globally [12] illustrate the increased prioritization of NTDs. Nevertheless, this is not the case for all diseases; for example, one interviewee (interview 2, academia) mentioned that for some NTDs, there are fewer powerful actors driving the international agenda. They said that some specific diseases such as Chagas are “still relying on a very reduced number of key actors. There are not many people who actually make a difference, and these are the same actors than ten years ago, basically” (interview 2, academia).

**Fig 2:**
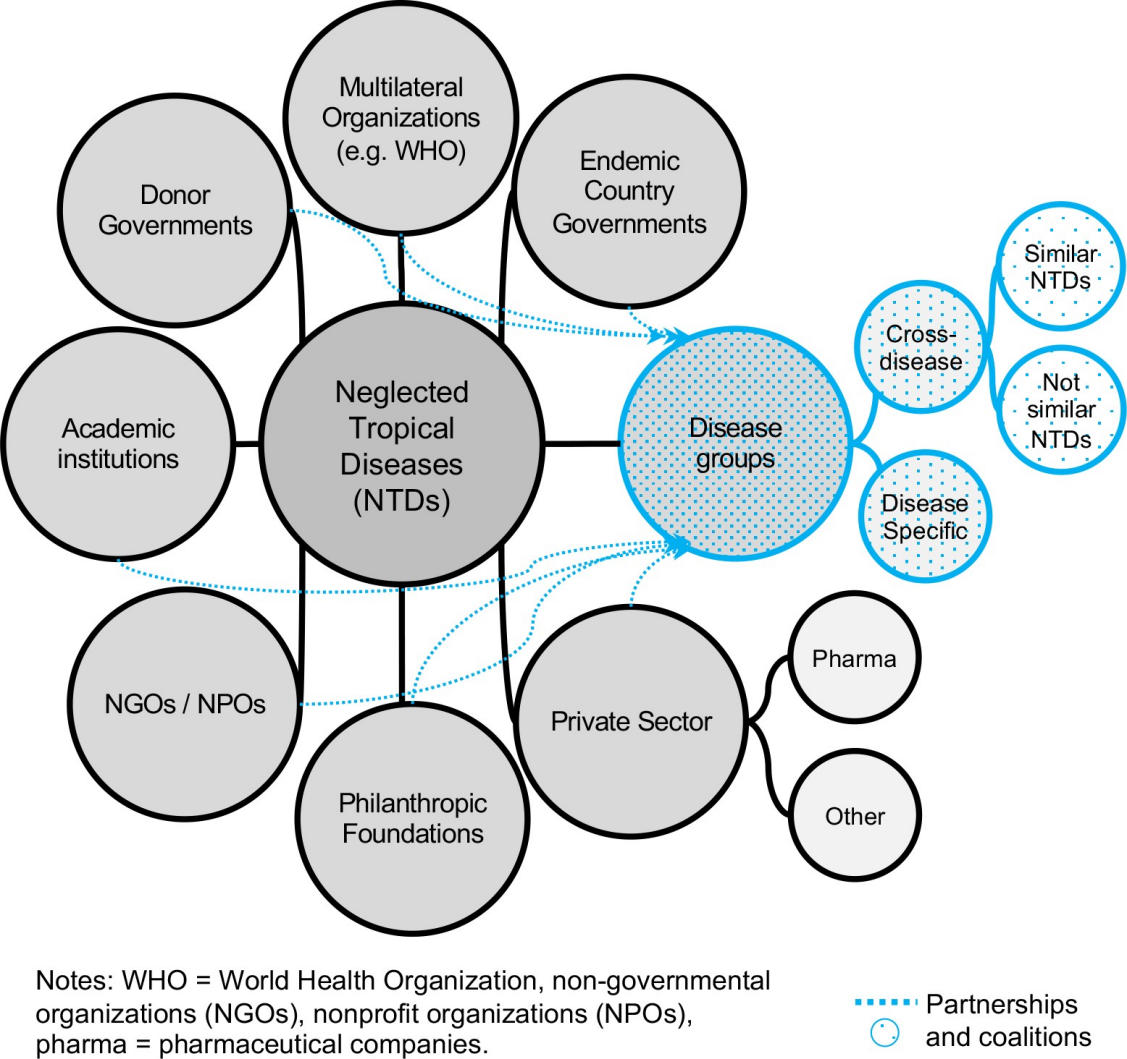
NTDs global actors network.

Data triangulated from the three sources used in this study revealed that at the global level, the NTD community comprises eight types of actors (see Fig 2). (1) Multilateral organizations where WHO leads collective actions; (2) Ministries of Health in endemic countries; (3) donor governments where the United States of America—U.S.A. (e.g. through USAID) and the government of the United Kingdom of Great Britain and Northern Ireland—U.K. (e.g. through the Department of International Development—DFID) lead in terms of support and funding; (4) academics representing academic institutions from around the globe; (5) non-governmental organizations (NGOs) (e.g. Médecins Sans Frontières) and nonprofit organizations (NPOs) (e.g. Drugs for Neglected Diseases initiative—DNDi); (6) philanthropic foundations such as the Bill & Melinda Gates Foundation; (7) private sector companies represented mostly by the pharmaceutical sector but also from other sectors, for example, the company TOMS provides shoes for the prevention and control of some NTDs (e.g. soil-transmitted helminths); and (8) disease groups.

Disease groups are in the form of partnerships and coalitions that comprise individuals from the other seven groups of actors. Disease groups are subcategorized in disease-specific groups and cross-disease groups. Disease-specific groups focus on a single disease (e.g. GAELF = Global Alliance for the Elimination of Lymphatic Filariasis). Cross-disease groups work on several diseases, some groups focus on diseases that have similarities in terms of their characteristics or types of interventions (e.g. Schistosomiasis Control Initiative—schistosomiasis and soil-transmitted helminthiasis), while other groups focus on a broader variety of NTDs (e.g. UK Coalition Against NTDs—all NTDs). These groups allow for actors from different sectors to converge for research, collaboration, and advocacy. One interviewee (interview 4, academia) mentioned that the purpose of cross-disease groupings varies. Some focus on making sure the targets are met, some on advocacy at the government level, and others on reporting research to influence funding priorities.

The role of actors within the community varies. One of the core functions of the WHO is “providing leadership on matters critical to health and engaging in partnerships where joint action is needed” [31], as well as establishing norms and standards, and facilitating technical support [31]. It is also responsible for ensuring NTD drugs donated by pharmaceutical companies reach the populations. The role of endemic country governments is fundamental to portray NTDs as a health priority and to ensure global policies are translated at the national level. Nevertheless, at the national level these diseases tend to not be prioritized, for example, by not having national NTD policies, in part due to a lack of understanding of their burden and consequences (interview 4, academia). An interviewee based in an endemic country, with experience working with varied stakeholders nationally and internationally, mentioned that beyond having global and national NTD targets, there is a need to make NTDs a daily priority of health executors (interview 10, NGO based in LMIC).

Donor governments and philanthropic foundations (e.g. Children’s Investment Fund Foundation, the END Fund) provide funding, for example, for medicines, R&D, and programming. Research outputs from academics and their recommendations help inform how money could be spent [32]. Academics are also involved in the delivery of interventions through research collaborations between or across disease groups. Civil society mobilization is high at a global level and low at the national level, according to remarks by some interviewees, non-governmental organizations (NGOs) are in this category.

International NGOs have played an important role in obtaining and providing data on the disease burden in endemic countries [33], as well as in fostering partnerships to respond to critical situations (interview 10, NGO based in LMIC). The data are used to advocate at the national and international level. One interviewee (interview 2, academia) who has worked with international NGOs in endemic and non-endemic countries mentioned that when these organizations leave a country, no evidence is collected and the diseases tend to be forgotten, placing great responsibility and dependence on these organizations.

Pharmaceutical companies are involved in drug donations but also in providing knowledge and building capacity in endemic countries to facilitate having a more significant impact with donated drugs, said one interviewee from the industry (interview 5, pharmaceutical company). Other interviewees mentioned benefits pharmaceutical companies obtain from donating; first, they are able to enter new markets and second, they improve their public reputation. Other companies from different sectors within the “private sector” category of actors, also collaborate but to a lesser extent expressed an interviewee (interview 4, academia).

The diversity of areas of expertise and focus of actors in the NTD community is one of the reasons for which partnerships are particularly valued. This community engages in public-private partnerships, product development partnerships, and private-private partnerships [30,34,35]. This shows and was confirmed during the interviews, that there is some cohesion among actors of the NTD community and that it has improved over the years, particularly among actors from the same category (e.g. pharmaceutical companies, disease-specific groups) or working on diseases with similar characteristics or interventions. Interviewees expressed that communication between NTD groups is not as fluid as that within groups, but nonetheless, it is also more fluid than during the pre-Millennium Development Goals (MDGs) period. The degree of cohesion of the NTD community is reflected in the following remarks:

“…there is some sort of sense of belonging among the NTD community. People like the hashtag NTDs, it’s been quite effective to bring together people who have an appetite for tropical medicine. I think it’s been successful in that sense…” (interview 3, international NGO operating in endemic and non-endemic settings)

### Ideas

The second category of the framework is ideas and focuses on how the actors involved with the issue understand and position it. The internal and external frames are the two factors included in this element [15]. Several paradigms and key arguments have been used to make NTDs resonate with different global agendas. The global political prioritization of these diseases has been raised around human rights [8,36,37], global public goods [23,36,38–40], socio-economic development [8,10,41], ethical and moral [13,40,42], and health economics perspectives [6,28,43,44]. Global policy documents and academic publications have built their arguments around the characteristics of these diseases causing and promoting poverty [6,10]; the cost-effectiveness of their interventions [44–46]; and the right to access to essential medicines [33,36,44]. Ignoring NTDs has been referred to as a moral failure [40]. The geographical overlap and co-morbidity between different NTDs is also part of the internal and external frames [20,42,45]. These arguments have been aligned with the global health and development agenda [10,43,47].

The NTD concept focuses on two things, the idea of neglect and the emphasis on diseases with similar characteristics and their impact on poverty and development [11,48–50]. Several interviewees stated that creating an NTD label has been fundamental to obtain global attention. NTD as a concept also has some negative aspects. One is that it encompasses diseases with different characteristics and modes of transmission, and the other that some diseases receive greater attention, particularly those using preventive chemotherapy. One interviewee expressed that the main failure has been that frequently when people refer to NTDs, they mean 5–7 diseases (interview 11, multilateral organization). This same person said that nevertheless, the “…NTD concept has saved a lot of lives”.

While several lists of NTDs exist, the WHO priority list [51] is the one leading global health priorities and facilitating access to funding from different actors. While some interviewees of this study stated that the criteria and process to be placed on that list was not so clear, in 2016, the NTD-STAG recommended the WHO to develop a process for inclusion of new diseases. One person suggested that the NTD category should be flexible so that diseases that are no longer neglected can leave to give space for diseases that are (interview 3, international NGO operating in endemic and non-endemic settings).

The Global Plan listed 20 priority diseases, but the number and types of diseases included in the WHO priority list has varied. Four diseases were removed from global priorities and were not included in the 1^st^ WHO NTD report, namely: anthroponotic leishmaniasis, anthrax, brucellosis, and Japanese encephalitis. From 2010 until early 2017 the WHO prioritized 17 NTDs. The current priority list includes 20 diseases, four that were not part of the Global Plan: foodborne trematode infections, mycetoma, scabies and other ectoparasites, and snakebite envenoming. Table 1 presents the evolution of NTDs prioritized by the WHO as reported in their global documents, reports, and website since the launch of the Global Plan.

**Table 1:** NTDs prioritized by WHO as reported in global documents and website.

Global Plan (2007)			Report 1 (2010)	Roadmap (2012)	Report 2 (2013)	Report 3 (2015)	Report 4 (2017)	WHO website (2018/19)
“Tool ready”	1. Dracunliasis	→	1. Dracunliasis (guinea-worm disease)	1. Dracunliasis (guinea-worm disease)	1. Dracunliasis (guinea-worm disease)	1. Dracunliasis (guinea-worm disease)	1. Dracunliasis	1. Dracunliasis (guinea-worm disease)
	2. Leprosy	→	2. Leprosy (Hansen disease)	2. Leprosy (Hansen disease)	2. Leprosy	Leprosy	2. Leprosy	2. Leprosy (Hansen disease)
	3. Lymphatic filariasis	→	3. Lymphatic filariasis	3. Lymphatic filariasis	3. Lymphatic filariasis	3. Lymphatic filariasis	3. Lymphatic filariasis	3. Lymphatic filariasis
	4. Anthroponotic leishmaniasis	**X**						
	5. Blinding trachoma	→	4. Trachoma	4. Blinding trachoma	4. Trachoma	4. Trachoma	4. Trachoma	4. Trachoma
	6. Cysticercosis	→	5. Cysticercosis	5. Cysticercosis	5. Taeniasis / cysticercosis	5. Taeniasis and (neuro)cysticercosis	5. *Taenia solium* taeniasis and neurocysticercosis	5. Taeniasis / Cysticercosis
	7. Echinococcosis	→	6. Echinococcosis	6. Echinococcosis	6. Echinococcosis	6. Echinococcosis	6. Cystic and alveolar echinococcosis	6. Echinococcosis
	8. Onchocerciasis	→	7. Onchocerciasis (river blindness)	7. Onchocerciasis	7. Onchocerciasis (river blindness)	7. Onchocerciasis (river blindness)	7. Onchocerciasis	7. Onchocerciasis (river blindness)
	9. Rabies	→	8. Rabies	8. Human dog-mediated rabies	8. Rabies	8. Rabies	8. Rabies	8. Rabies
	10. Schistosomiasis	→	9. Schistosomiasis (bilharziasis)	9. Schistosomiasis (bilharziasis)	9. Schistosomiasis	9. Schistosomiasis	9. Schistosomiasis	9. Schistosomiasis
	11. Soil-transmitted helminthiasis	→	10. Soil-transmitted helminthiases	10. Soil-transmitted helminthiases (intestinal worms)	10. Soil-transmitted helminthiases	10. Soil-transmitted helminthiases	10. Soil-transmitted helminthiases	10. Soil-transmitted helminthiases
	12. Yaws	→	11. Endemic treponematoses	11. Endemic treponematoses (yaws)	11. Endemic treponematoses	11. Endemic treponematoses	11. Endemic treponematoses (yaws)	11. Yaws (Endemic treponematoses)
“Tool Deficient”	13. Anthrax	**X**						
	14. Brucellosis	**X**						
	15. Buruli Ulcer	→	12. Buruli ulcer (*Mycrobacterium ulcerans* infection)	12. Buruli Ulcer	12. Buruli ulcer	12. Buruli ulcer	12. Buruli ulcer	12. Buruli ulcer
	16. Chagas disease	→	13. Chagas disease (American trypanosomiasis)	13. Chagas disease	13. Chagas disease	13. Chagas disease	13. Chagas disease	13. Chagas disease
	17. Dengue	→	14. Dengue	14. Dengue	14. Dengue	14. Dengue	14. Dengue and other arbovirus-related diseases	14. Dengue and Chikungunya
	18. Human African Trypanosomiasis	→	15. Human African trypanosomiasis (sleeping sickness)	15. Human African trypanosomiasis (sleeping sickness)	15. Human African trypanosomiasis (sleeping sickness)	15. Human African trypanosomiasis (sleeping sickness)	15. Human African trypanosomiasis	15. Human African trypanosomiasis (sleeping sickness)
	19. Japanese Encephalitis	**X**						
	20. Leishmaniases	→	16. Leishmaniasis	16. Leishmaniasis	16. Leishmaniasis	16. Leishmaniasis	16. Leishmaniasis	16. Leishmaniasis
			17. Foodborne trematode infections*	17. Foodborne trematode infections*	17. Foodborne trematodiases*	17. Foodborne trematodiases*	17. Foodborne trematodiases*	17. Foodborne trematodiases*
							18. Mycetoma*	18. Mycetoma, chromoblastomycosis and other deep mycoses *
								19. Scabies and other ectoparasites*
								20. Snakebite envenoming*

Symbol description: → Moved as a priority after the Global Plan, X did not move beyond the Global Plan, * Not included in the Global Plan

The Global Plan originally classified NTDs in two groups of “tool-ready” or “tool-deficient” diseases. The former focused on diseases having low-cost and available strategies (e.g. preventive chemotherapy), and the latter on diseases with strategies more difficult and expensive to implement (e.g. innovative and intensified disease management). Two of the interviewees mentioned that this initial grouping of NTDs created an imbalance in the allocation of resources and funds that focused predominantly on “tool-ready” diseases, especially on those controlled by preventive chemotherapy (1—academia, 11—multilateral organization). One of them stated that for this reason, the tool-ready and tool-deficient terminology was abandoned, although its effects persist today (interview 1, academia).

The external portrayal of NTDs as a problem by the NTD community is also aligned with the global health agenda; this is seen for example in references to primary health care (PHC) [4,10,44], universal health care (UHC) [4,10,30,35,44], and health systems strengthening [13,35,47]. The latter was frequently mentioned by some of the actors interviewed. Consistency between the UHC targets with those of NTDs has been postulated in global documents, including in WHA 66.12 on NTDs [52], and in academic publications [23,35]. NTD control and elimination has been suggested to be a “litmus test” or a measure for success of UHC in reaching the most vulnerable, this was repeatedly postulated in the 3^rd^ WHO report on NTDs [44]. NTD strategies and targets are viewed as essential elements and milestones on the path leading to UHC [34,44].

Another part of the framing and key arguments that have been used by the NTD community to attract attention has been the possibility of elimination or eradication of some of these diseases [24,51]. The use of these words has been attractive to donors as expressed by an interviewee from academia who stated:

“…you will never get rid of diabetes or hypertension; you can just mitigate, perhaps decrease the incidence to some extent, to have early diagnosis, to promote early management so that you can prevent complications, but there is no spectacular objective like elimination or eradication, and that is a very appealing aspect of NTDs.” (interview 1, academia)

In addition to the paradigms and key arguments used, the portrayal of NTDs as a category of diseases has evolved over the years. When Kenneth Warren introduced the term Great Neglected Diseases in 1977 [53], he referred to “great” in terms of prevalence and “neglected” in relation to the lack of international funding and support for research they had at the time [53]. Other terms used in the documents and articles reviewed include: communicable diseases, neglected infections, infectious and parasitic diseases, neglected diseases, neglected tropical infections, and diseases of poverty. In 2003 during the 29th G8 Summit held in Évian-les-Bains, France, these diseases were referred to as “diseases affecting mostly developing countries (‘neglected diseases’)” [54]. These diseases were referred for the first time as “neglected tropical diseases” in a meeting of NTD actors held in Berlin in 2005 [48]. The first time they were referred as such by the G8 was in 2008 at the Hokkaido Toyako 34^th^ Summit [55,56].

“…at the beginning the idea for us was to think about diseases, tropical diseases for neglected population…But you can imagine that was not politically correct, it was impossible to tell to the different ministries that they were neglecting their populations… so we kept the idea of neglect because it was quite important for us because it was affecting a reality…so we started to build this concept of neglect for tropical diseases, and we started to mobilize different people who was interested by that and we made the first meeting in Berlin…” (interview 11, multilateral organization)

### Political context

The environments surrounding the policy actors are referred to by Shiffman and Smith [15] as the political context. Two factors are used in this category, the presence of policy windows or moments when conditions from the environment align in favor of an issue, and the global governance structure that provides a platform for action [15]. The period before the new millennium was characterized by the lack of funding and research focused on NTDs, the lack of available medicines for the most vulnerable in the developing world, and by the lack of involvement and coordination of actors of different sectors within the NTDs community [11,28]. According to some of the articles reviewed [33,36,38] and to comments from some interviewees (1—academia, 3—international NGO operating in endemic and non-endemic settings), at that period of time the pharmaceutical industry acted according to a market-driven rationale.

In the year 2000, the United Nations launched the Millennium Declaration. This global instrument delineated seven global targets; three focused on health; one of this was to “combat HIV/AIDS, malaria and other diseases” [9]. For the NTD community, the “other diseases” category included NTDs [8,13,41]. Being categorized as “other diseases” was the tipping point to mobilize and unite the community [20]. Given the targets and population focus of the MDGs, the absence of NTDs from this agenda was not considered acceptable [8,20,41]. This situation presented an important political moment to group and align the NTD community.

Global meetings were convened by key champions from the WHO, academia, and civil society organizations, that resulted in significant changes in the governance of NTDs. Two meetings held in Berlin in 2003 and 2005 co-organized by WHO, the German Ministry for Economic Cooperation and Development, the German Ministry for Health and Social Security, the German Technical Cooperation (GTZ), Kreditanstalt für Wiederaufbau (KFW), and the Special Programme for Research and Training in Tropical Diseases (TDR); helped to make the argument for the need of increasing resources for NTDs, of making low-cost and effective interventions, and for looking for their possible integration and co-implementation [48,50].

**Fig 3:**
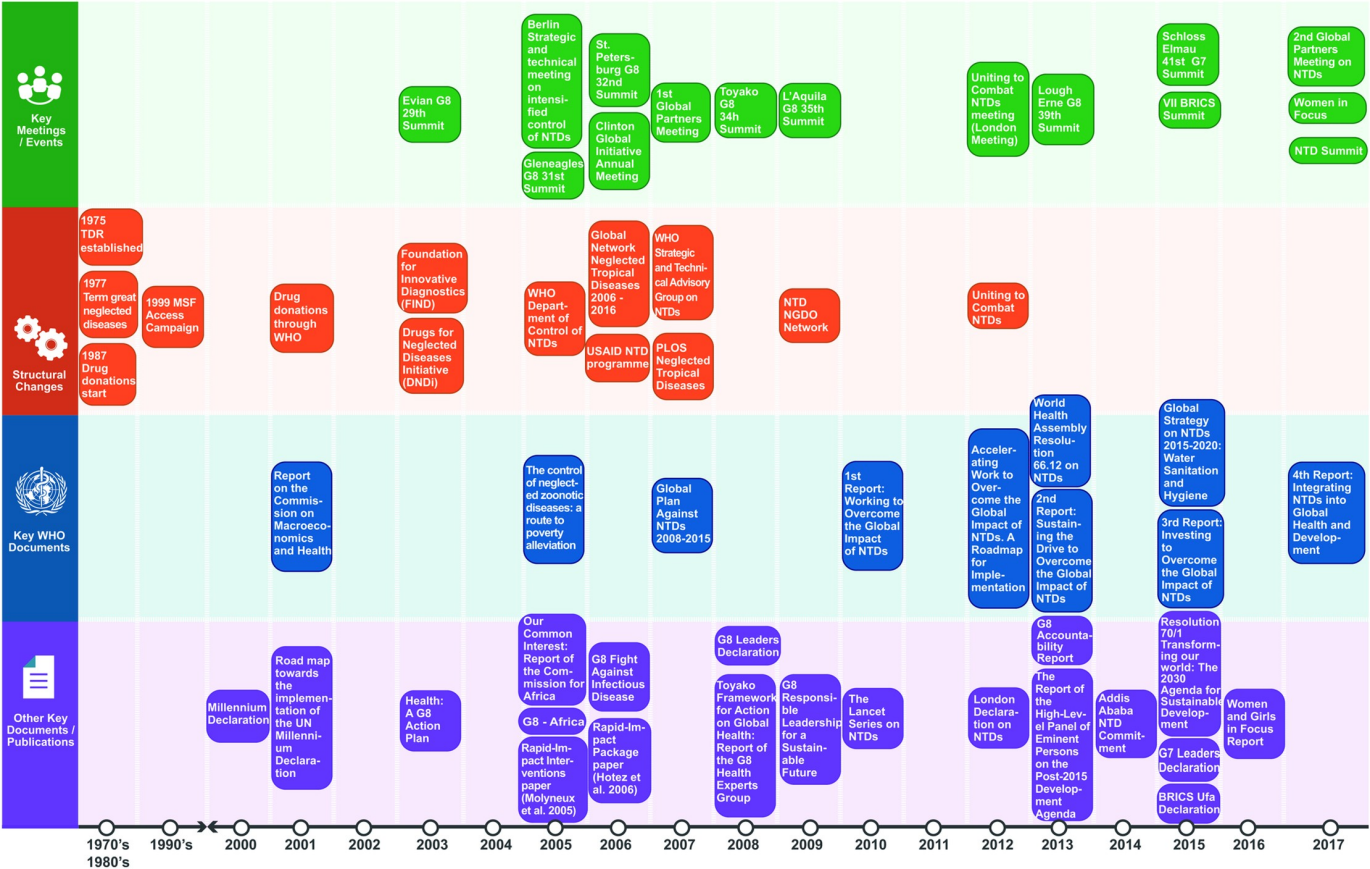
Key milestones in the global policy agenda for NTDs 1970s - 2017.

NTD awareness and prioritization were grounded on global policy documents such as the 2001 Report on the Commission on Macroeconomics and Health [57], and the 2005 report of the Commission for Africa [58], as well as academic papers [33,36,38,45,46]. Two initiatives were created in 2003: the Foundation for Innovative New Diagnostics (FIND) and the Drugs for Neglected Diseases Initiative (DNDi). Two years later, the WHO Department of Control of NTDs was established. In 2006 the USAID NTD program was created as well as the Global Network for Neglected Tropical Diseases; the later lasted until 2016 and focused on raising awareness and advocating for NTDs at the global and national levels [25]. See these and other structural changes in Fig 3.

Efforts from the Global Network for Neglected Tropical Diseases and other actors included advocating for the inclusion of NTDs in the 2030 Agenda for Sustainable Development, as well as on the G7 [59] and BRICs [60] priorities. In relation to these political groups, an interviewee mentioned that NTDs have been portrayed at the G7 and G20:

“… The G7 and G20 platforms are difficult platforms to achieve important progress. The G7 has raised the profile and political commitment to NTDs, specifically at several times. More recently, through the G7 2015 Leaders Declaration, which provided commitment to working on NTD treatment and R&D. But the difficult nature of those platforms is how that is translated into actual policy change and funding commitments.” (interview 6, NGO)

This person also mentioned that NTDs have yet to reach the BRICS platform and that countries pertaining to this political group focus more on mutual collaboration rather than on providing funding.

In 2007 the WHO Neglected Tropical Diseases Strategic and Technical Advisory Group (NTD-STAG) and the first journal fully dedicated to these conditions, PLOS NTDs, were launched. This same year, the WHO convened the first global partners meeting on NTDs assembling about 200 actors from different sectors, calling for increasing the priority of NTD control in the global public health agenda [61]. Another milestone reached in 2007 was the release of the first global plan for NTDs covering the period 2008–2015 [62]. An interviewee who participated in building the momentum for NTDs expressed that the vision of NTDs and its strategy were built around access to diagnosis, to treatment, to the health structure, and to health services; and that the two priorities the WHO had for NTDs were to create a new concept and to find solutions to increase access (interview 11, multilateral organization).

Having a more cohesive community and a label to help portray the burden of these diseases as a group, served as a stepping stone for reaching consensus on strategies and targets. The first WHO report on NTDs presented an analysis of the causes, enabling factors, and strategic interventions to combat these diseases, and of how addressing these conditions contributed to development targets [10].

In 2012 the NTD Roadmap was launched setting targets until 2020 [51]. This document gave continuity to the NTD Global Plan and leveraged on the first WHO Report. The roadmap linked NTDs with UHC. This same year, a meeting entitled “Uniting to Combat NTDs: Ending the Neglect and Reaching the 2020 Goals” [11] convened global health actors from different sectors to form a global coalition to focus on 10 NTDs. This initiative, focused predominantly on diseases controlled with preventive chemotherapy, was formalized with the endorsement of the London Declaration by pharmaceutical companies, the U.S.A., U.K. and the United Arab Emirates (U.A.E.) governments, the Bill & Melinda Gates Foundation, the World Bank, and other global health organizations [63]. These actors agreed to provide more than US$785 million to NTDs [63], and this partnership has been essential to sustain, expand, and extend the supply of drugs and drug access programs [24]. Together, the London meeting and the London Declaration are considered a “global health turning point” [11]. In words of an interviewee:

“…I think that is important that every stakeholder is recognized for the fantastic work they are doing, and without the combination of this collaboration we will not get that…So that’s the London Declaration and it’s been, in my mind, interesting to see and is fascinating to see that before 2012 there were no collaborations across pharma industry on addressing NTDs…now I have to say, that we realized that we weren’t speaking to one another, and we need to speak to one another…” (interview 8, pharmaceutical company)

Another policy milestone reached during this decade was in 2013 with the release of the first WHA resolution (WHA 66.12) on NTDs as a group of diseases rather than as single diseases. This resolution also made an explicit call to support the Member States with funding, in the implementation of national plans, and in the promotion of UHC. During that year, as well as in 2015 and 2017, new WHO reports were released on NTDs.

A global policy window opened with the discussions about the MDG post-2015 agenda. Conversations within the NTD community encompassed ways to ensure NTDs were included in this agenda [64]. In 2012, a High-Level Panel of Eminent Persons on the Post-2015 Development Agenda was assigned to make recommendations. This group developed a report released in 2013 where for the first time, a global health target for NTDs was included [65]. An interviewee who participated in these processes expressed that engaging with key individuals involved in shaping the sustainable development bill using the argument of “what can be done for little money”, was essential to have NTDs included in the targets (interview 7, academia).

According to one of the interviewees, the periods before the financial commitments of funders expire, have been essential in NTD advocacy (interview 6, NGO). They said that civil society organizations use the years preceding the end of funding to motivate renewal and increase financial commitment to these diseases by governments and large funders. An example of this is the policy momentum generated by the NTD community in 2017, before the period of expiration of the NTD Roadmap that set targets until 2020.

The second global partners meeting on NTDs was held in 2017 and convened health ministries, donors, philanthropists, industry representatives, civil society organizations, and academics to pledge additional support for these diseases beyond the 2020 Roadmap targets. Announcements made at this meeting included a £360 million investment by the U.K. Government (for five years), pledges of €25 million by the Government of Belgium (until 2025), grants of US$335 million by the Bill & Melinda Gates Foundation (until 2020); and medicine pledges by organizations from Argentina, Brazil, and Japan, as well as of traps for the control of tsetse flies by a Swiss company [29]. In this meeting, the 4^th^ WHO report on NTDs was launched [12]. During this same week, the NTD Summit 2017 organized by the Uniting to Combat NTDs coalition took place. These key milestones are summarized in Fig 3 (see Fig 3).

Concerning the global governance of the NTD community, the documents and articles reviewed revealed that for diseases included in the list of WHO priorities, the main instrument delineating and guiding collective actions is the NTD Roadmap [51], supported by the WHA 66.12. No evidence was found about the presence of norms to guide strategies for NTDs not prioritized by the WHO. In terms of guiding institutions, overall, the analysis of documents showed that WHO is recognized as the leading institution, but the other NTD community actors (see Fig 2) also participate actively in shaping the global agenda. Comments from some of the interviewees revealed mixed perceptions and experiences about the institutional positioning of WHO in relation to the other actors. Opinions of the interviewees included:

“…people like to compare WHO for example with NGO, there’s no commonality, it’s not made for the same thing and the legitimacy of decisions etcetera is WHO, because they are working for the governments” (interview 11, multilateral organization)
“…Currently, WHO is a little bit sidelined by other actors such as the big funders and coalitions of funders…” (interview 2, academia)
“…although we interact very closely with WHO, which is the one that is supposed to [be] guiding the global policy, it turns out that it is the interactions we have with the countries, the money at which the countries are using, the technologies that we are working on, that ends up guiding global policy, which should be the other way around” (interview 9, NPO)

### Issue characteristics

The final category is issue characteristics, which denotes the problem’s attributes in relation to three factors: the presence of credible indicators, the severity or size of the burden, and the existence of effective interventions [15]. In the documents and articles reviewed, the burden of NTDs has been portrayed in relation to their DALYs, the funds allocated to them, the populations affected, and the geographical regions where these diseases are endemic. Due to the low mortality and high morbidity of most NTDs, DALYs are used to portray their burden [21]. This is often used to compare the NTD burden with that of HIV/AIDS, malaria, and tuberculosis (TB). This comparison is a strategy used for advocacy to gain attention by further reinforcing the message that the burden of disease is high. Debates around the burden assigned to NTDs have been constant within the NTD community since the 1990s, and several DALYs calculations are used to portray the issue [2,35,66]. For example, that of the 2010 global burden of disease (GBD) study which assigned a burden related to NTDs of 26.1 million DALYs [3], and that of another study that calculated a burden of 47.9 million DALYs [2]. The latter study included 17 NTDs prioritized by the WHO and other NTDs not included in their list of priorities [2].

Linkages between the burden and the resources allocated to NTDs have been described. For example, a commonly cited reference made in the reviewed articles was that of a study by Liese and Schubert [67] who found that despite the burden and the affected populations, from 2003 to 2007 official development assistance (ODA) for health only allocated 0.6% for NTDs.

The documents and articles reviewed also show that the NTD burden has been described in terms of the populations affected (e.g. bottom billion, publics with little political power) [6,8,13,28], geographic and economic characteristics of the settings where these diseases are endemic (e.g. developing world, low and middle-income (LMIC) countries, G20 countries) [4,10,20,21,68], as well as of their climate characteristics (e.g. tropical and subtropical settings) [22,40]. The epidemiological transition from low-middle to higher-income economies (e.g. United States, European Union, Australia) is sometimes referred to as being related to external factors such as population mobility and climate change [30,40]. Nonetheless, regardless of the geographic location, the vulnerability of the populations affected has always been maintained in the description of features of NTDs [4,44].

Concerning how NTDs have been presented and by whom, one interviewee mentioned that the arguments used to drive resources to NTDs have been aligned by the interests of specific groups with the power to influence policymaking. They stated:

“The organizations with power are related to the North…and they are who say what problems are important to our countries, regrettably…There is a group of North American and European experts who say what tropical diseases are important. Something that you can imagine that exacerbates my spirit as a [nationality] and there are a bunch of diseases that they want to include because they are working on them and want to prioritize them, because they know that the money goes that way and not in other diseases.” (interview 12, academia, LMIC based)

When describing the NTD problem, two other areas emerged from the documents and articles reviewed. The first one related to gender and the second one to non-communicable diseases (NCDs). About gender, some of the documents mentioned that gender differences in exposure to risk factors, the burden of care, access to health-care, and other social consequences (e.g. stigma) exist [4,10,44,69]. Some of the articles revealed that the linkages between NTDs and gender have been scarcely portrayed in global policies [21,35]. Two of the individuals interviewed also posited that gender has still not permeated the NTD global agenda. Likewise, in relation to the interrelationship between the burden of NTDs with that of non-communicable diseases (NCDs), direct associations between these diseases were made in some of the global policy documents and articles reviewed [21,30,70,71], such as the following: “Mostly unappreciated is the stealth contribution of the NTDs to the NCD disease burden in developing countries” [21].

The determinants under issue characteristics also include effective interventions and the availability of indicators to monitor progress. Based on the literature, the rapid-impact package to simultaneously control seven NTDs (i.e. ascariasis, trichuriasis, hookworm, lymphatic filariasis, onchocerciasis, schistosomiasis, trachoma) with four medicines (i.e. albendazole, azithromycin, ivermectin, and praziquantel) at a cost of about US$0.40 per person per year [45,46] was used to portray a solution for the NTD burden. The terms used to position this intervention included “quick fix approach” [43], “low-hanging fruit” [72], and “quick wins” [47], given that three of the medicines were donated by pharmaceutical companies and one was available at a low cost [8,45].

While the Global Plan focused on 20 diseases and presented nine strategic areas for action that guided the NTD community in raising the profile of these conditions globally, the 1^st^ WHO report presented a group of interventions to address these conditions. These interventions have since been promoted and were included in the NTD Roadmap. The five interventions also referred as public health strategies, are: preventive chemotherapy and transmission control (PCT), innovative and intensified disease management (IDM), vector ecology and management (VEM), veterinary public health measures, and the provision of safe water, sanitation and hygiene (WASH). The names of these interventions have had minor changes over the years (see Table 2).

**Table 2:** Priority Interventions for NTDs as Documented in the WHO Roadmap and Reports.

No.	Report 1 (2010)	Roadmap (2011)	Report 2 (2013)	Report 3 (2015)
1	Preventive chemotherapy	Preventive chemotherapy	Preventive chemotherapy	Preventive chemotherapy
2	Intensified case-management	Intensified **case-detection** and case management	I**nnovative and** intensified disease-management	Innovative and intensified disease management
3	Vector control	Vector **and intermediate host** control	Vector control **and pesticide management**	Vector **ecology and management**
4	Veterinary public health	Veterinary public health **at the human-animal interface**	Veterinary public-health **services using the one-health concept**	Veterinary public-health **services**
5	Provision of safe water, sanitation and hygiene	Provision of safe water, sanitation and hygiene	**Safe drinking-water, basic sanitation and hygiene services, and education**	Provision of safe water, sanitation and hygiene

The NTD Roadmap presents the milestones and targets against which the NTD community measures success in reducing the global burden of these diseases. Progress is reported in the WHO Weekly Epidemiological Record, the WHO NTD reports, and also through the Uniting to Combat NTDs scorecard to track the diseases included in the London Declaration [30,73].

## Discussion

This study examined the determinants from a policy perspective that have led to the prioritization of NTDs on the global health policy agenda. These diseases moved from having a low presence on the global health policy agenda and being absent from the MDGs, to receiving higher recognition and being included in the SDGs. Not being on the MDG agenda was the main condition that elicited the mobilization of a group of actors that initiated processes to raise the priority of these diseases globally. The Shiffman and Smith framework [15] provided a structure for analysis that enabled the identification of factors that resulted in the gradual prioritization of NTDs during the past decade. The utility of the framework of actor power, ideas, political contexts, and issue characteristics is demonstrated in this study.

Concerning actor power, the presence of committed individuals and institutions facilitated engagement with other actors from different sectors, which in turn resulted in increased collaborations. With respect to ideas, overall, the NTD community has shown consistency in the portrayal of the problem, causes, and solutions. The use of terminology (e.g. cost-effectiveness, eradication, elimination) that resonated with the perspectives and arguments of different groups has shown to be successful in attracting attention and funding. The creation of a label that clearly and explicitly denotes the characteristics of the diseases, and that results in a higher disease burden than having these as individual diseases, has shown to be one of the key elements for success in gaining external recognition.

Regarding political context, the NTD community has used policy windows present in the environment, but has also created favorable policy moments such as meetings of global partners to generate or sustain commitment from global actors. A governance structure lead by the WHO with strong participation of actors from multiple sectors has been favorable in gaining recognition for these diseases. With respect to issue characteristics, the NTD community has consistently presented the severity of the problem and the interventions needed to address them, backed up with scientific evidence. The existence of clear common goals to drive collective action and monitor progress has also been beneficial.

Challenges revealed in this study include making a case for diseases that were once described as “tool-deficient”, lacking simple and cost-effective tools and treatment. So far, diseases that were previously categorized as “tool-ready” received greater attention, and so has the main intervention used to address them, specifically preventive chemotherapy and transmission control (PCT). Although the importance of PCT is undeniable and should be maintained, other priority interventions are necessary. Likewise, diseases prevented, controlled, or managed with other priority strategies have not been prioritized to the same extent as diseases using PCT, even though they are included in the WHO list of priority NTDs; of these diseases zoonotic NTDs are notedly sidelined. Diseases not included in the WHO priority list are even more neglected. A possible way to make a case for diseases and needs of the NTDs community that are not currently prioritized would be to involve more actors from the Global South in determining global priorities.

Identifying differences and similarities with the experiences of other health issues using the Shiffman and Smith framework [15] is also pertinent. Unlike the case of NTDs, some other health issues have fragmented communities [15,17,18] and lack global governance structures [15,17], limiting collaboration and the ability to reach consensus on how to present the issues and arguments for investment. Difficulties in measuring the burden have also hampered global prioritization of some health issues [15,18]. Similarly to NTDs, communities working on building the priority of global surgery and NCDs also mobilized to be incorporated in the post-2015 agenda [18,19], and the NCD community also used the economic argument as part of framing the issue [19]. In relation to how health issues and their potential solutions were framed, although NTD interventions focus on control and NCD interventions on prevention, a common strategy implemented by these two communities was to group different diseases under a same label. This strategy has proven to be successful for NTDs, but not necessarily for NCDs [19].

Sustaining and embracing new partnerships should continue to be a priority for the NTD community. While involving actors from multiple sectors differentiates NTDs from other health issues [22,35,74], it is recommended that the areas of expertise and contribution of these actors be expanded. Possible ways include developing programs such as those of drug donations [75,76] but with a focus on the other priority interventions. As many of these interventions require structural changes at the national or local level, the involvement of more actors from endemic countries is paramount to foster collaboration and drive actions [35].

The 2030 Agenda for Sustainable Development calls for integrated responses [14]. Because NTDs cut across all SDGs [34], increasing their global prioritization can catalyze progress to achieve global targets. For example, evidence from this study shows that NTDs are a “litmus test” for UHC (SDG target 3.8) and that partnerships (SDG 17) have been essential to mobilize the NTD community towards common targets. Priority interventions such as WASH and VEM are also aligned with the SDG agenda (Goals 6 and 11), and because the populations affected by these diseases are predominantly vulnerable, combating NTDs is directly in line with the goals of ending poverty (SDG 1) and inequalities (SDG 10).

In the pathway to achieving the SDGs, the WHO launched a global consultation in 2019 to help determine the priorities of the new Roadmap for NTDs (2021–2030). Discussions on the new NTD Roadmap should make a stronger case of how addressing NTDs contributes to attaining UHC priorities, ensuring the new agenda prioritizes strengthening health systems and integrates NTD policies with those of other intersecting issues such as gender and NCDs. The new NTD Roadmap should provide a platform for intersectoral and inter-programmatic collaborations (e.g. human–animal health) that are currently challenging to implement. Based on findings from this study, other areas requiring the attention of the NTD community include implementing measures to increase the engagement of other actors such as the G20, BRICs, endemic countries, and civil society in endemic countries.

### Strengths and limitations

Findings from this study should be seen in the light of some strengths and limitations. Strengths include the innovation in using the Shiffman and Smith policy framework [15] for the first time to examine the process of prioritization of NTDs in the global health agenda. Another strength is the use of three sources of information, including official documents, peer-reviewed publications, and direct knowledge of individuals involved in prioritizing NTDs. Limitations include the limited sample size of interviewees, and that the fourth report on NTDs [12] was not included because it was published after the document analysis was complete. Furthermore, academic publications were not collected in a systematic way, meaning that some aspects might have been missed from the academic literature. An additional limitation is that the framework used does not allow to determine the relative power of some actors over others; this could be considered for future studies and new versions of the framework.

## Conclusion

The determinants of political priority help understand the processes that have led to the current position of NTDs in the global policy agenda. The presence of individuals and institutions leading collective action, the involvement of actors representing different sectors, the creation of the NTD label as well as the framing of the problem and possible solutions, and the use of key environmental moments, have influenced in building the priority of these diseases once excluded from global health and development priorities. Despite the successes reached to date, NTDs remain neglected. Continuing to leverage policy windows and create moments to sustain commitment, along with greater involvement of governments and civil society of endemic countries are of paramount. Further, more attention should be placed on former “tool-deficient” diseases and to neglected NTDs, such as zoonoses or those not currently prioritized by the WHO. Involving more actors from endemic countries in determining global NTD priorities could help in setting responses that are relevant to the needs of health systems and affected populations. The findings from this study can help develop strategies to drive actions to implement the goals of the new Roadmap for NTDs, in the pathway to UHC and sustainable development.

## Supporting information

S1 Text List of questions for interviews. (DOCX)

S2 Text Documents and articles reviewed. (DOCX)
