# Supplementary material for: The process of building the priority of neglected tropical diseases: A global policy analysis
Source: PLoS Negl Trop Dis. 2020 Aug 12;14(8):e0008498. doi: 10.1371/journal.pntd.0008498 (PMC7423089; doi:10.1371/journal.pntd.0008498)
Supplement: S1 Text — (DOCX) [file pntd.0008498.s001.docx]

**S1. List of questions for interviews**

The following questions serve as a database from which the interviewers will select some and will adapt them based on the profile of each interviewee.

1. What has been your role in formulating or contributing to the development of the policy agenda for NTDs?
2. What political factors have influenced in setting the NTDs policy agenda? Where there any changes in the norms and institutions of the health sector that provided a platform for collective action?
3. What economic factors have driven the interest in NTDs at the global policy level?

Probe: Relationship of poverty with NTDs and development

1. What do you consider are the key NTDs global policy documents?
2. The list of WHO priority NTDs includes some zoonotic diseases, how do you see the scene for these diseases?
3. How have these documents considered/framed UHC and PHC?
4. How is gender positioned in NTDs policies?
5. Each NTD WHO report has focused in different “themes”, the 4^th^ report now mentions development. How do these “themes” reflect the evolution of NTDs policies and how do they relate to the global context?
6. How were the WHO 17 priority NTDs selected and by whom?
7. What is the process to include a disease in the list?

Probe: What evidence is needed?

Probe: Who takes part in the process?

1. What does it mean to be one of the included NTDs in the WHO list?

Probe: How does being in the list relate to access to funding?

1. How were the 10 NTDs of the London Declaration selected?

Probe: Why do they not focus on the same 17 diseases as WHO if the Declaration intends to support the WHO Roadmap for the 17 NTDs?

1. What does not being in the London Declaration priority NTDs mean for the excluded diseases?
2. When we read the policies and the reports, we find there are 5 strategies/interventions to prevent, control, eliminate and eradicate NTDs. What can you tell us about them?

Probe: How were they selected?

Probe: Why in reports 1-2 they are called strategies whereas in reports 3-4 they are called interventions?

1. Most (if not all) NTDs benefit from WASH interventions, and there is a Global Strategy for NTDs and WASH 2015-2020. How was the process to develop this policy? What lessons does it provide to promote intersectoral and inter-programmatic collaboration with other areas such as veterinary public health?

Probe: Do they share common resources?

1. Many NTDs policy documents mention the importance of health education and behavior change promotion, what can you tell us about this?
2. Do you see commonalities in the policy agenda between NTDs and NCDs?
3. How were NTDs policies developed?

Probe: How were they initiated?

Probe: How were they negotiated?

Probe: How were they financed?

1. What facilitated the development of the NTDs global policies?

Probe: Where there any policy windows that served as enablers?

Probe: Was there evidence that facilitated the process?

1. Where there any actors who served as champions?

Probe: How were they involved? What did they do?

1. What facilitated the dissemination of the NTDs global policies?

Probe: What actors participated in this and how?

Probe: Where were these policies disseminated?

Probe: How were these policies disseminated?

1. What barriers were presented along the way?

Probe: Where there other diseases with highest priority at the policy level? If so, which diseases?

Probe: How was/is the evidence base supporting the need to create policies about NTDs?

Probe: Where there any actors who did not facilitate setting the agenda?

1. What are some lessons learned from the NTDs policy agenda setting?

Probe: how could we use these lessons to incorporate more diseases in the list of priority NTDs?

Probe: How could LMICs learn from these lessons to advocate the creation of NTDs national policies?

1. Which actors played a key role in shaping the NTDs policy(ies)?

Probe: How have they participated and why them?

1. How did these actors first began to work for NTDs?

Probe: How were they engaged?

Probe: Who and what helped to involve them?

1. Why does your organization work in NTDs?

Probe: Since when does it work in NTDs?

1. In what NTDs does your organization support or work on?

Probe: Why?

Probe: How were these diseases selected?

1. In relation to NTDs, with which other organizations do you work?

Probe: Is your organization part of any alliance, coalition, group?

1. How does the relationship between WHO and [interviewee’s organization] work?
2. How do you see the future participation of your organization in advocating for NTDs?
3. How does the relationship between the *WHO Department of Control of Neglected Tropical Diseases* and *the Strategic and Technical and Advisory Group for NTDs (STAG-NTDs)* work?
4. How does *WHO Department of Control of Neglected Tropical Diseases* work with Uniting to Combat NTDs?
5. How do the different Uniting to Combat NTDs *Stakeholders Working Groups (SWG)* relate to the WHO *Strategic and Technical and Advisory Group for NTDs (STAG-NTDs)?*
