# Supplementary material for: The process of building the priority of neglected tropical diseases: A global policy analysis
Source: PLoS Negl Trop Dis. 2020 Aug 12;14(8):e0008498. doi: 10.1371/journal.pntd.0008498 (PMC7423089; doi:10.1371/journal.pntd.0008498)
Supplement: S2 Text — (DOCX) [file pntd.0008498.s002.docx]

**S2. List of documents and articles reviewed**

| **No.** | **Reference** |
| --- | --- |
| *Global documents* | |
| 1 | G8. G8 Communiqué Okinawa 2000. Kyushu-Okinawa G8 26th Summit. 2000; published online July 23. http://www.mofa.go.jp/policy/economy/summit/2000/documents/index.html (accessed Aug 11, 2016). |
| 2 | Ministry of Foreign Affairs, Japan. Japan’s Initiative in the Fight against Infectious and Parasitic Diseases on the occasion of the Kyushu-Okinawa G8 Summit (‘Okinawa ID (Infectious diseases) Initiative’). Ministry of Foreign Affairs of Japan. 2000; published online July. http://www.mofa.go.jp/policy/oda/summit/infection.html (accessed Aug 11, 2016). |
| 3 | G8. Health: A G8 Action Plan. Evian G8 29th Summit. 2003; published online June 2. http://www.g8.utoronto.ca/summit/2003evian/health_en.html (accessed July 26, 2016). |
| 4 | G8. Africa. Gleneagles G8 31st Summit. 2005; published online July 8. http://www.g8.utoronto.ca/summit/2005gleneagles/africa.html (accessed July 26, 2016). |
| 5 | G8. Fight Against Infectious Disease. St. Petersburg G8 32nd Summit. 2006; published online July 16. http://www.g8.utoronto.ca/summit/2006stpetersburg/infdis.html (accessed July 26, 2016). |
| 6 | World Health Organization. Report of the First Meeting of WHO Strategic and Technical Advisory Group on Neglected Tropical Diseases. Geneva, Switzerland, 2007. |
| 7 | World Health Organization. Global Plan to Combat Neglected Tropical Diseases 2008–2015. 2007. |
| 8 | World Health Organization. Report of the Global Partners’ Meeting on Neglected Tropical Diseases. A Turning Point 2007. Geneva, Switzerland, 2007. |
| 9 | G8. G8 Hokkaido Toyako Summit Leaders Declaration. Hokkaido Toyako G8 34th Summit. 2008; published online July 8. http://www.mofa.go.jp/policy/economy/summit/2008/doc/doc080714__en.html (accessed Aug 11, 2016). |
| 10 | G8 Health Experts Group. Toyako Framework for Action on Global Health: Report of the G8 Health Experts Group. Hokkaido Toyako G8 34th Summit. 2008; published online July 8. http://www.g8.utoronto.ca/summit/2008hokkaido/index.html (accessed July 26, 2016). |
| 11 | G8. G8 Leaders Declaration: Responsible Leadership for a Sustainable Future. L’Aquila G8 35th Summit. 2009; published online July 8. http://www.g8.utoronto.ca/summit/2009laquila/ (accessed July 26, 2016). |
| 12 | World Health Organization. Report of the WHO Strategic and Technical Advisory Group on Neglected Tropical Diseases (STAG-NTD). Geneva, Switzerland, 2009. |
| 13 | G8. Muskoka Declaration: Recovery and New Beginnings. Muskoka G8 36th Summit. 2010; published online June 26. http://www.g8.utoronto.ca/summit/2010muskoka/communique.html (accessed July 26, 2016). |
| 14 | G8. Muskoka Accountability Report. Assessing action and results against development-related commitments. Muskoka, Canada, 2010. |
| 15 | World Health Organization. Working to Overcome the Global Impact of Neglected Tropical Diseases. First WHO Report on Neglected Tropical Diseases. 2010. |
| 16 | World Health Organization. Report of the WHO Strategic and Technical Advisory Group on Neglected Tropical Diseases (STAG-NTD). Geneva, Switzerland, 2010. |
| 17 | G8. Deauville Accountability Report. G8 Commitments on Health and Food Security: State of Delivery and Results. Deauville, France, 2011 http://www.g8.utoronto.ca/summit/2011deauville/road.html (accessed July 26, 2016). |
| 18 | World Health Organization. Report of the WHO Strategic and Technical Advisory Group on Neglected Tropical Diseases. Geneva, Switzerland, 2011. |
| 19 | G8. Camp David Accountability Report. Actions, Approach and Results. Maryland, United States, 2012. |
| 20 | Uniting to Combat Neglected Tropical Diseases. The London Declaration on Neglected Tropical Diseases. 2012. http://unitingtocombatntds.org/resource/london-declaration (accessed July 26, 2016). |
| 21 | World Health Organization. Accelerating work to overcome the global impact of neglected tropical diseases a roadmap for implementation. 2012. |
| 22 | World Health Organization. Report of the WHO Strategic and Technical Advisory Group on Neglected Tropical Diseases. Geneva, Switzerland, 2012. |
| 23 | G8. Lough Erne accountability report. Keeping our promises. United Kingdom, 2013. |
| 24 | Uniting to Combat Neglected Tropical Diseases. From Promises to Progress: The First Annual Report on the London Declaration on NTDs. 2013. |
| 25 | World Health Organization. Sustaining the Drive to Overcome the Global Impact of Neglected Tropical Diseases. Second WHO Report on Neglected Tropical Diseases. Geneva, Switzerland, 2013. |
| 26 | World Health Organization. Report of the sixth meeting of the WHO Strategic and Technical Advisory Group on Neglected Tropical Diseases. Geneva, Switzerland, 2013. |
| 27 | Uniting to Combat Neglected Tropical Diseases. Delivering on Promises and Driving Progress: The Second Report on Uniting to Combat NTDs. 2014. |
| 28 | Uniting to Combat Neglected Tropical Diseases. The Addis Ababa NTD Commitment. 2014. http://unitingtocombatntds.org/resource/addis-ababa-ntd-commitment (accessed July 25, 2016). |
| 29 | World Health Organization. Report of the WHO Strategic and Technical Advisory Group on Neglected Tropical Diseases. Geneva, Switzerland, 2014. |
| 30 | G7. Leaders’ Declaration G7 Summit 7-8 June 2015. Germany: Schloss Elmau G7 41st Summit, 2015. |
| 31 | Global Network for Neglected Tropical Diseases. Open Letter to G7 Heads of State on Neglected Tropical Diseases. 2015; published online March 4. http://www.globalnetwork.org/g7-and-g20 (accessed July 25, 2016). |
| 32 | Uniting to Combat Neglected Tropical Diseases. Country Leadership and Collaboration on Neglected Tropical Diseases. Third progress report of the London Declaration. 2015. |
| 33 | World Health Organization. Investing to Overcome the Global Impact of Neglected Tropical Diseases. Third WHO Report on Neglected Tropical Diseases. 2015. |
| 34 | World Health Organization. Report of the WHO Strategic and Technical Advisory Group on Neglected Tropical Diseases. Geneva, Switzerland, 2015. |
| 35 | World Health Organization. Water Sanitation and Hygiene for Accelerating and Sustaining Progress on Neglected Tropical Diseases. A global Strategy 2015-2020. 2015. |
| 36 | Uniting to Combat Neglected Tropical Diseases. Reaching the unreached. Fourth progress report of the London Declaration. 2016. |
| 37 | World Health Organization. Report of the WHO Strategic and Technical Advisory Group on Neglected Tropical Diseases. Geneva, Switzerland, 2016. |
| *World Health Assembly Resolutions* | |
| 1 | World Health Assembly. Resolution 54.19 Schistosomiasis and soil-transmitted helminth infections. World Health Assembly, 2001. |
| 2 | World Health Assembly. Resolution 55.17 Prevention and control of dengue fever and dengue hemorrhagic fever. World Health Assembly, 2002. |
| 3 | World Health Assembly. Resolution 56.26 Elimination of avoidable blindness. World Health Assembly, 2003. |
| 4 | World Health Assembly. Resolution 56.7 Pan African Tsetse and trypanosomiasis eradication campaign. World Health Assembly, 2003. |
| 5 | World Health Assembly. Resolution 57.9 Eradication of dracunculiasis. World Health Assembly, 2004. |
| 6 | World Health Assembly. Resolution 57.1 Surveillance and control of Mycobacterium ulcerans disease (Buruli ulcer). World Health Assembly, 2004. |
| 7 | World Health Assembly. Resolution 57.2 Control of human African trypanosomiasis. World Health Assembly, 2004. |
| 8 | World Health Assembly. Resolution 59.25 Prevention of avoidable blindness and visual impairment. World Health Assembly, 2006. |
| 8 | World Health Assembly. Resolution 60.13 Control of leishmaniasis. World Health Assembly, 2007. |
| 10 | World Health Assembly. Resolution 62.1 Prevention of avoidable blindness and visual impairment. World Health Assembly, 2009. |
| 11 | World Health Assembly. Resolution 63.2 Chagas disease: control and elimination. World Health Assembly, 2010. |
| 12 | World Health Assembly. Resolution 64.16 Eradication of dracunculiasis. World Health Assembly, 2011. |
| 13 | World Health Assembly. Resolution 64.24 Drinking-water, sanitation and health. World Health Assembly, 2011. |
| 14 | World Health Assembly. Resolution 65.21 Elimination of schistosomiasis. World Health Assembly, 2012. |
| 15 | World Health Assembly. Resolution 66.12 Neglected tropical diseases. World Health Assembly, 2013. |
| *Articles* | |
| 1 | Pécoul B, Chirac P, Trouiller P, Pinel J. Access to Essential Drugs in Poor Countries: A Lost Battle? *JAMA* 1999; **281**: 361–7. |
| 2 | Webber D, Kremer M. Perspectives on stimulating industrial research and development for neglected infectious diseases. *Bull World Health Organ* 2001; **79**: 735–41. |
| 3 | Trouiller P, Olliaro P, Torreele E, Orbinski J, Laing R, Ford N. Drug development for neglected diseases: a deficient market and a public-health policy failure.  *Lancet* 2002; **359**: 2188–94. |
| 4 | Pécoul B. New Drugs for Neglected Diseases: From Pipeline to Patients. *PLOS Medicine* 2004; **1**: e6. |
| 5 | Fenwick A, Molyneux D, Nantulya V. Achieving the Millennium Development Goals. *Lancet* 2005; **365**: 1029–30. |
| 6 | Molyneux DH, Hotez PJ, Fenwick A. “Rapid-Impact Interventions”: How a Policy of Integrated Control for Africa’s Neglected Tropical Diseases Could Benefit the Poor. *PLoS Med* 2005; **2**: e336. |
| 7 | Hotez PJ, Molyneux DH, Fenwick A, Ottesen E, Sachs SE, Sachs JD. Incorporating a Rapid-Impact Package for Neglected Tropical Diseases with Programs for HIV/AIDS, Tuberculosis, and Malaria. *PLoS Med* 2006; **3**: e102. |
| 8 | Sachs JD, Hotez PJ. Fighting Tropical Diseases. *Science* 2006; **311**: 1521–1521. |
| 9 | Hotez PJ, Molyneux DH, Fenwick A, *et al.* Control of Neglected Tropical Diseases. *N Engl J Med* 2007; **357**: 1018–27. |
| 10 | Molyneux DH. Combating the ‘other diseases’ of MDG 6: changing the paradigm to achieve equity and poverty reduction? *Trans R Soc Trop Med Hyg* 2008; **102**: 509–19. |
| 11 | Butler D. Neglected disease boost. *Nature* 2009; **457**: 772–3. |
| 12 | Hotez PJ, Fenwick A, Savioli L, Molyneux DH. Rescuing the bottom billion through control of neglected tropical diseases. *Lancet* 2009; **373**: 1570–5. |
| 13 | Liese BH, Schubert L. Official development assistance for health–how neglected are neglected tropical diseases? An analysis of health financing. *Int Health* 2009; **1**: 141–7. |
| 14 | Molyneux DH. Neglected tropical diseases—beyond the tipping point? *Lancet* 2010; **375**: 3–4. |
| 15 | Kirby T. David Molyneux: raising the profile of neglected tropical diseases. *Lancet* 2010; **375**: 21. |
| 16 | Liese B, Rosenberg M, Schratz A. Programmes, partnerships, and governance for elimination and control of neglected tropical diseases. *Lancet* 2010; **375**: 67–76. |
| 17 | Gyapong JO, Gyapong M, Yellu N, *et al.* Integration of control of neglected tropical diseases into health-care systems: challenges and opportunities. *Lancet* 2010; **375**: 160–5. |
| 18 | Baker M, Mathieu E, Fleming F, *et al.* Mapping, monitoring, and surveillance of neglected tropical diseases: towards a policy framework. *Lancet* 2010; **375**: 231–8. |
| 19 | Conteh L, Engels T, Molyneux DH. Socioeconomic aspects of neglected tropical diseases. *Lancet* 2010; **375**: 239–47. |
| 20 | Hotez PJ, Pecoul B. “Manifesto” for Advancing the Control and Elimination of Neglected Tropical Diseases. *PLoS Negl Trop Dis* 2010; **4**: e718. |
| 21 | Molyneux DH, Malecela MN. Neglected Tropical Diseases and the Millennium Development Goals-why the ‘other diseases’ matter: reality versus rhetoric. *Parasit Vectors* 2011; **4**: 234. |
| 22 | Hotez PJ, Goraleski KA. Neglected Tropical Diseases and the 2012 US Presidential Election. *PLoS Negl Trop Dis* 2011; **5**: e1431. |
| 23 | Molyneux DH. The ‘Neglected Tropical Diseases’: now a brand identity; responsibilities, context and promise. *Parasit Vectors* 2012; **5**: 23. |
| 24 | Nagpal S, Sinclair D, Garner P. Has the NTD Community Neglected Evidence-Based Policy? *PLoS Negl Trop Dis* 2013; **7**: e2238. |
| 25 | Hotez PJ. NTDs V.2.0: “Blue Marble Health”—Neglected Tropical Disease Control and Elimination in a Shifting Health Policy Landscape. *PLoS Negl Trop Dis* 2013; **7**: e2570. |
| 26 | Molyneux DH. Neglected tropical diseases: now more than just ‘other diseases’— the post-2015 agenda. *Int Health* 2014; **6**: 172–80. |
| 27 | Keating C. Ken Warren and the Rockefeller Foundation’s Great Neglected Diseases Network, 1978–1988: The Transformation of Tropical and Global Medicine. *Mol Med* 2014; **20**: S24–30. |
| 28 | Mableson HE, Okello A, Picozzi K, Welburn SC. Neglected Zoonotic Diseases—The Long and Winding Road to Advocacy. *PLoS Negl Trop Dis* 2014; **8**: e2800. |
| 29 | Molyneux DH. Eradication and elimination: facing the challenges, tempering expectations. *Int Health* 2015; **7**: 299–301. |
| 30 | Molyneux DH, Savioli L, Engels D. Neglected tropical diseases: progress towards addressing the chronic pandemic. *Lancet* 2017; **389**: 312–25. |
| 31 | Hotez P, Bundy DAP. The PLOS Neglected Tropical Diseases decade. *PLoS Negl Trop Dis* 2017; **11**: e0005479. |
| 32 | Iltis AS, Matthews KRW. NTD policy priorities: Science, values, and agenda setting. *PLoS Negl Trop Dis* 2017; **11**: e0005431. |
| 33 | Fürst T, Salari P, Llamas LM, Steinmann P, Fitzpatrick C, Tediosi F. Global health policy and neglected tropical diseases: Then, now, and in the years to come. *PLoS Negl Trop Dis* 2017; **11**: e0005759. |
| 34 | Molyneux DH. The London Declaration on Neglected Tropical Diseases: 5 years on. *Trans R Soc Trop Med Hyg* 2016; **110**: 623–5. |
| 35 | Bangert M, Molyneux DH, Lindsay SW, Fitzpatrick C, Engels D. The cross-cutting contribution of the end of neglected tropical diseases to the sustainable development goals. *Infect Dis Poverty* 2017; **6**: 73. |
| 36 | Hotez PJ. Ten failings in global neglected tropical diseases control. *PLoS Negl Trop Dis* 2017; **11**: e0005896. |
| 37 | Molyneux DH, Dean L, Adekeye O, Stothard JR, Theobald S. The changing global landscape of health and disease: addressing challenges and opportunities for sustaining progress towards control and elimination of neglected tropical diseases (NTDs). *Parasitology* 2018; **145**: 1647–54. |
| 38 | Hotez PJ, Fenwick A, Ray SE, Hay SI, Molyneux DH. “Rapid impact” 10 years after: The first “decade” (2006–2016) of integrated neglected tropical disease control. *PLoS Negl Trop Dis* 2018; **12**. DOI:10.1371/journal.pntd.0006137. |

Notes: * = The Lancet 2010 Series on NTDs
